# Heterogeneous nuclear ribonucleoprotein M associates with mTORC2 and regulates muscle differentiation

**DOI:** 10.1038/srep41159

**Published:** 2017-01-20

**Authors:** Wei-Yen Chen, Chia-Lung Lin, Jen-Hua Chuang, Fu-Yu Chiu, Yun-Ya Sun, Mei-Chih Liang, Yenshou Lin

**Affiliations:** 1 Department of Life Science, National Taiwan Normal University, Taipei 116, Taiwan; 2 Department of Biological Science and Technology, National Chiao Tung University, Hsinchu 300, Taiwan

## Abstract

Mammalian target of rapamycin (mTOR) plays a range of crucial roles in cell survival, growth, proliferation, metabolism, and morphology. However, mTOR forms two distinct complexes, mTOR complex 1 and mTOR complex 2 (mTORC1 and mTORC2), via association with a series of different components; this allows the complexes to execute their wide range of functions. This study explores further the composition of the mTORC2 complex. Utilizing Rictor knock-out cells, immunoprecipitation and mass spectrometry, a novel Rictor associated protein, heterogeneous nuclear ribonucleoprotein M (hnRNP M), was identified. The association between hnRNP M and Rictor was verified using recombinant and endogenous protein and the binding site was found to be within aa 1~532 of hnRNP M. The presence of hnRNP M significantly affects phosphorylation of SGK1 S422, but not of Akt S473, PKCα S657 and PKCζ T560. Furthermore, hnRNP M also plays a critical role in muscle differentiation because knock-down of either hnRNP M or Rictor in C2C12 myoblasts reduced differentiation. This decrease is able to be rescued by overexpression SGK S422D in hnRNP M knockdown C2C12 myoblasts. Taken together, we have identified a novel Rictor/mTOR binding molecule, hnRNP M, that allows mTORC2 signaling to phosphorylate SGK1 thus regulating muscle differentiation.

mTOR responds to a variety of different stimuli, including growth factors, amino acid levels and energy deprivation. In addition to the effects of growth factors, nutrients and stress, both the Hippo and WNT pathways have been recently investigated in relation to mTOR and it has been shown that these pathways participate in the regulation of mTOR kinases^1^,^2^. Although mTOR itself consists of a single gene only, the signaling relays involving this gene has been found to act in two distinctly different ways, named via mTOR complex 1 (mTORC1) and via mTOR complex 2 (mTORC2). The first of these complexes, namely mTORC1, consists of the proteins Raptor and LST8 with Raptor functioning as a scaffolding protein that couples mTOR with the substrates S6K and 4E-BP1. Furthermore, the Tuberous Sclerosis complex, a heterodimer of TSC1 and TSC2, has been found to act as a negative regulator of mTOR signaling via its ability to serve as a GTPase activator that affects a Ras-like small GTPase called Rheb^3^. The TSC1/2 complex is a major site of regulation of the insulin pathway and is also associated with energy depletion via AMP activated protein kinase (AMPK). A more recent investigation concerning mTORC1 has revealed that the TSC1-TSC2-TBC1D7 (TSC-TBC) complex is a functional complex that senses specific cellular growth conditions and this complex possesses Rheb-GAP activity too^4^. In addition to the above, pro-autophagic UNC-51-like kinase 1 (ULK1) has been identified as an mTORC1 substrate and has been shown to be essential for autophagosome formation^5^.

The alternative complex, which is made up of mTOR, Rictor, mSin1 and mLST8, is named mTORC2^6^. This complex does not include Raptor. Less is known about the upstream pathways, the regulation and the roles of mTORC2 compared to mTORC1. After the identification of Akt serine 473 (S473) as an mTORC2 substrate^7^, other AGC kinases (protein kinase A/G/C) were also identified as additional substrates. Sarbassov *et al*. showed that PKCα phosphorylation at a hydrophobic motif and the activity of the phosphorylated form are dependent on mTORC2^6^. Furthermore, mTORC2 appears to phosphorylate SGK1 at serine 422 (S422) in response to growth factors, even though SGK1 lacks a pleckstrin homology (PH) domain and is able to be activated independent of membrane recruitment^8^. Growth factors, via the PI3K pathway, also have been found to stimulate intrinsic mTORC2 kinase activity. Recently, it has been found that PKCζ, which lacks phosphorylation sites in its hydrophobic motif, is also a specific and direct substrate of mTORC2; this interaction results in the phosphorylating of threonine 560 (T560) within the turn motif of PKCζ^9^. At the organism level, both TORC2 and the mTORC2 complex have been shown to regulate actin assembly^10^. In NIH 3T3 cells, insulin and lysophosphatidic acid (LPA) are able to promote lamellopodia and stress fiber formation respectively, whereas serum is able to bring about both responses. These actin responses are completely resistant to rapamycin, but are inhibited by depletion of Rictor, but not depletion of Raptor. The result is a decrease in the membrane anchoring of cortical actin and paxillin, as well as a reduction in paxillin tyrosine phosphorylation^6^. Furthermore, the muscles of Rictor knockout mice have been found to exhibit decreased insulin-stimulated glucose uptake and these mice also show glucose intolerance^11^. The broad trend in terms of scientific findings seems to suggest that both the downstream signaling, as well as biological function, of mTORC2 are worthy of further in-depth exploration.

During the present study we used a series of different approaches, including immunoprecipitation and mass spectometry analysis, and found a novel Rictor associated protein, namely hnRNP M. Our detailed findings on the relationship between this protein and the mTORC2 complex, together with the role of the complex in muscle differentiation, helps to shed light on the field of mTORC2 signaling.

## Results

### Identifying hnRNP M as a component of the mTORC2 complex by Rictor immunoprecipitation (IP)

**Figure 1:**
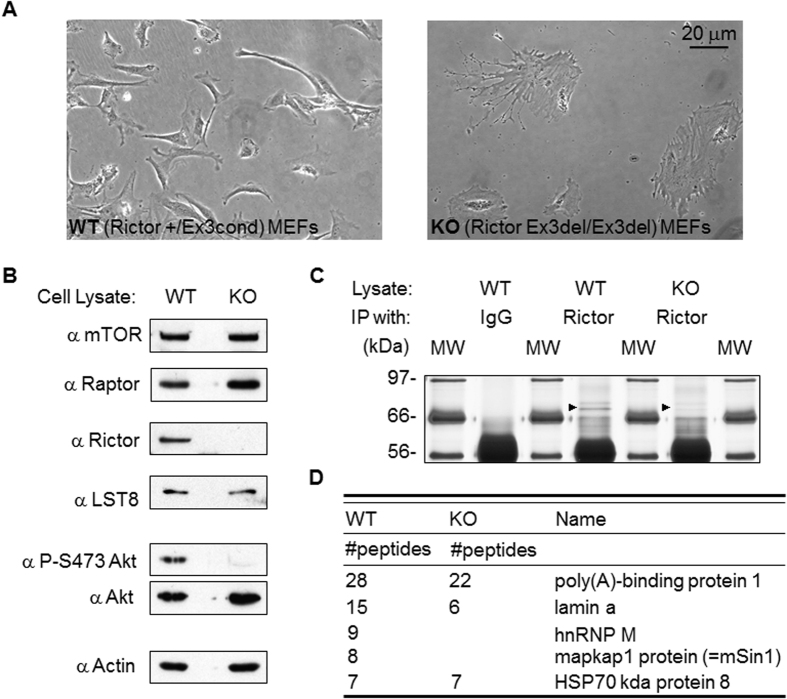
Characterization of Rictor knock-out mouse embryonic fibroblasts (MEFs) and the discovery of hnRNP M. (**A**) Exon 3 of Rictor was targeted for deletion using a Cre/flox approach. The cellular morphology of the MEF cells with either the Rictor wild-type or Rictor knock-out genotypes were observed and recorded. (**B**) Examination of the relevant mTORC1 and mTORC2 components in the Rictor WT and KO MEFs. The phosphorylation of Akt Ser473 was assessed in order to verify the loss-of-function of Rictor. (**C**) Silver staining of a SDS-PAGE gel that was used to separate the anti-Rictor antibody IP components present in the Rictor WT and Rictor KO MEF cells. Total protein from each cell lysate (1 mg) was subjected to IP using 2 μg of Rictor antibody or rabbit normal IgG for each IP. The samples were separated by 8% SDS-PAGE, which was followed by silver staining of the gel. The bands with a molecular weight of around 70 kD were sent for analysis and are indicated by arrows. (**D**) The proteins identified by mass spectrometry analysis of the bands are listed. Both mSin1 and hnRNP M were found to be present in the IP results using Rictor WT MEFs, but were absent in the IP results using Rictor KO MEFs.

The Rictor knock-out (KO) mouse embryonic fibroblast (MEF) cells were a generous gift from Dr. Magnuson of Vanderbilt University, USA. The appearance of the Rictor KO MEFs is very abnormal and branching is easily observed (Fig. 1A). Lysates obtained from the wild-type and knock-out Rictor MEFs were used to verify the efficacy of the Rictor knock-out. The results showed that the Rictor protein, as well as the Akt S473 signal, had disappeared as expected (Fig. 1B). These lysates were then subjected to immunoprecipitation (IP) using anti-Rictor antibody. The resulting IP complex was intensively washed, which was followed by separation by SDS-PAGE of the proteins that formed the complex. The bands obtained from the WT/Rictor IP were compared to the bands obtained from the WT/IgG IP and the KO/Rictor IP (Fig. 1C). Bands only present in the WT/Rictor IP lane, but clearly absent from the WT/IgG IP and KO/Rictor IP lanes were excised, purified and sent for mass spectrometry analysis (Supplementary Fig. S1). This approach was used with the aim of eliminating the majority of false positive candidates that would have overwhelmed the mass spectrometry analysis. As shown in the Fig. 1D, among the bands with a molecular weight of about 70 kD, this region being marked with a triangle in Fig. 1C, there were a few protein bands that fulfilled our selection criteria; these were found to include mSin1 and hnRNP M. The association between mSin1 and Rictor has been reported previously^12^,^13^, however an association between hnRNP M and Rictor is believed to be a novel finding.

### Verification of the association between hnRNP M and Rictor/mTORC2 using both recombinant protein and endogenous protein

**Figure 2:**
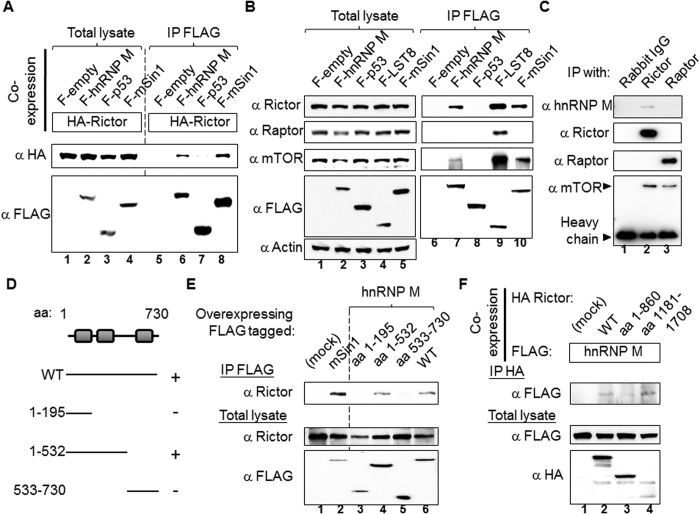
HnRNP M associates with Rictor, but not Raptor and the binding site is within the hnRNP M aa 1–532 region, but not within either the aa 1–195 or aa 533–730 regions. (**A**) HEK 293T cells were co-transfected with the indicated FLAG or HA tagged plasmids. The cell lysates were subjected to FLAG antibody IP. Anti-FLAG and anti-HA antibodies were used to assess the protein expression levels in the total lysates and in the IP samples. FLAG alone (F-empty) and FLAG p53 (F-p53) were included as negative controls, while FLAG mSin1 (F-mSin1) was included as a positive control. (**B**) HEK 293T cells were transfected with the indicated FLAG tagged plasmids only. The FLAG LST8 IP (F-LST8) was included as a control to assess the mTORC1/2 components present. Rictor, Raptor, and mTOR antibodies were used to classify the mTOR complexes. (**C**) Rictor, Raptor, and control antibodies were used to IP the various individual proteins in HEK 293T lysates. The heavy chain at the bottom of panel indicates the relative abundance of the antibodies used in the IP. (**D**) Full length and fragments of hnRNP M were used in the binding assay. HnRNP M contains three RNA recognition motifs (RRMs), which respectively are present at aa 71–144, aa 204–276, and aa 653–724. The recovery of the endogenous Rictor from the IP using FLAG-hnRNP M fragments is indicated on the right. (**E**) HEK 293T cells were transfected with plasmids encoding FLAG-tagged hnRNP M wild-type, aa 1–195, aa 1–532 and aa 533–730. FLAG alone and FLAG-mSin1 IP were included as controls. After FLAG antibody IP, the samples were processed and analyzed by Western blotting using Rictor antibody. All the presented blots are representative of one experiment that was repeated three times. (**F**) HEK 293T cells were co-transfected with the indicated FLAG-hnRNP M and HA tagged Rictor proteins (wild-type, aa 1–860 and 1181–1708). The lysates were subjected to HA antibody IP. Anti-FLAG and anti-HA antibodies were used to detect the relevant proteins in the total lysates and the IP samples. The presented blots are representative of one experiment that was repeated twice.

The hnRNP M gene was cloned into a mammalian expression vector. We established a number of positive controls before verifying the association between hnRNP M and Rictor/mTORC2. As shown in Fig. 2A, FLAG tagged hnRNP M is able to be co-immunoprecipitated with HA tagged Rictor in HEK 293T cells (lane 6 *vs*. lane 5). In the same experiment, FLAG-p53/HA-Rictor and FLAG-mSin1/HA-Rictor were respectively included as a negative control and a positive control. Furthermore, when we examined the association by comparing recombinant protein with endogenous protein, as shown in Fig. 2B; the experimental procedures used were similar to Fig. 2A except for two specific differences. Firstly, various antibodies recognizing the individual endogenous proteins were used rather than just anti-HA antibody. Secondly, we included FLAG-LST8 as an extra positive control. The results show that hnRNP M is associated with mTORC2 since FLAG-hnRNP M was found to associate with endogenous Rictor and with mTOR, but not with Raptor, although the mTOR binding level is quite low. There was also proof of binding using a reciprocal IP, namely a hnRNP M IP, as well as a Rictor IP; these results of which are presented in Fig. 1C. Finally, the most compelling evidence supporting the presence of hnRNP M in the mTORC2 complex was obtained from the experiments where only endogenous proteins are analyzed. As shown in Fig. 2C, endogenous hnRNP M can be detected via an endogenous Rictor IP, but not via an endogenous Raptor IP. A rabbit normal IgG IP was used as the negative control.

### HnRNP M aa 1–532, but not HnRNP M aa 1–196 or HnRNP M aa 533–730, is involved in the association with Rictor

Mapping the binding domains of a protein has important implications; these include helping to determine details of the binding mechanism, showing how specific activators/inhibitors may function and helping with the development of drug targets. Based on the analysis by SMART (http://smart.embl-heidelberg.de/), hnRNP M contains three RNA recognition motifs (RRMs) (Fig. 2D). The RRM1, RRM2, and RRM3 are respectively present at aa 71–144, aa 204–276, and aa 653–724. There is also an arginine/methionine/glycine rich region in the region aa 400–612. Therefore, genes containing a series of fragments covered different RRM motifs were constructed. We found that hnRNP M aa 1–532 was able to associate with endogenous Rictor *in vivo*, but that neither hnRNP M aa 1–196 nor hnRNP M aa 533–730 were able to bind to Rictor (Fig. 2E). By comparing the results shown in Fig. 2E, we suggest that hnRNP M aa 196–532 is likely to contain the domain that associates with Rictor. Unfortunately we are unable to express hnRNP M aa 196–532 in the mammalian cells and this was also not possible using a bacterial expression system. Hence, our results indicate that hnRNP M aa 1–532 and most likely hnRNP M aa 196–532 is the region involved in binding to Rictor. Furthermore, we also characterized the part of Rictor that is able to bind to hnRNP M. Since there are no known domains/regions in Rictor, which has full length 1708 amino acids, of particular interest we constructed three different Rictor proteins that consisted of aa 1–860, aa 861–1300, and aa 1181–1708. However, fragment aa 861–1300 could not be expressed and therefore we only analyzed two fragments, namely the N-terminal fragment and the C-terminal fragment. As shown in the Fig. 2F, the result suggests that hnRNP M is associated much more strongly with C-terminal than N-terminal of Rictor.

### Depletion of hnRNP M decreases the insulin-induced phosphorylation of SGK1 at S422

**Figure 3:**
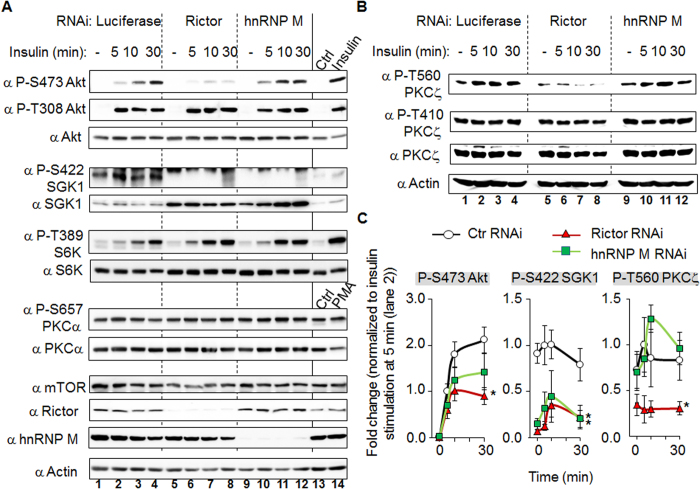
The hnRNP M knockdown affects phosphorylation of SGK1 S422 in a similar manner to that of Rictor knockdown, but does not affect other mTORC2 substrates such as PKCα. (**A**) C2C12 myoblasts cells containing RNAi targeting luciferase, Rictor or hnRNP M were stimulated using insulin 100 nM for various time intervals (5, 10, or 30 min) as indicated. The cell lysates were then processed and Western blot analysis performed. The last two lanes (lane 13, 14) were used as the antibodies efficacy controls. (**B**) Each cell lysate was treated and is arranged as outlined in the previous experiments except that blotting of the membranes was carried out using antibodies targeting total PKCζ or the phosphorylated form of PKCζ. (**C**) The level of protein phosphorylation present on these blots was quantified using NIH Image J freeware. The y-axis is the fold change expressed by normalizing against insulin stimulation at 5 min (panel A, lane 2), which was set as 1. All blots are representative of one experiment that was repeated four times. Data are the mean ± SD (n = 4). *p < 0.05 *vs*. luciferase RNAi at the same timing.

The direct substrates of mTORC2 have been established to be Akt S473^7^, SGK1 S422^8^ and PKCζ T560^9^. We next examined whether hnRNP M is involved in the phosphorylation of one or more of these substrates. It is worth noting that the anti-SGK1 S422 antibody purchased from Santa Cruz Biotechnology has been previously reported to recognize phosphorylated endogenous S6K1 and/or S6K2 (both about 70 kDa in size) much more effectively, based on immunoblot analysis, than phosphorylated endogenous SGK1^8^. Therefore it became necessary to examine carefully the signal around 50 kDa when studying the endogenous proteins. As shown in the Fig. 3A, several kinases within the insulin signaling cascade were activated; these findings are based on the increased phosphorylation of Akt S473, Akt T308, SGK1 S422, and S6K T389. These events could be detected after insulin stimulation for 5, 10, and 30 min (lane 1–4). Depletion of hnRNP M was able to decrease the phosphorylation of SGK1 S422, while at the same time there was no effect on Akt S473 or PKCα S657 phosphorylation. The samples in lane 13 and 14 were prepared as follows. C2C12 cells without virus infection were subjected to serum starvation for 4 h and this was followed by treatment with 100 nM insulin for 0 (lane 13) and 60 min (lane 14). Thus we used some known stimuli to treat the cells before Western blot analysis in order to verify the authenticity and specificity of the antibodies used. Furthermore, recently, PKCζ T560 has been shown to be a direct substrate of mTORC2^9^. We found that, while this particular site shows a reduced phosphorylation in the Rictor knockdown cells, nevertheless PKCζ T560 phosphorylation was not affected by hnRNP M knock-down (Fig. 3B). The quantitative results for the most important molecules, namely Akt S473, SGK1 S422, and PKCζ T560, are summarized in Fig. 3C. The effect of Rictor knockdown on SGK1 S422 correlates well with the effect of hnRNP M knockdown. These results imply that hnRNP M is likely to be involved in the Rictor/mTORC2 axis that regulates phosphorylation of SGK1 S422.

### Overexpression of hnRNP M increases the phosphorylation of SGK1 at S422

**Figure 4:**
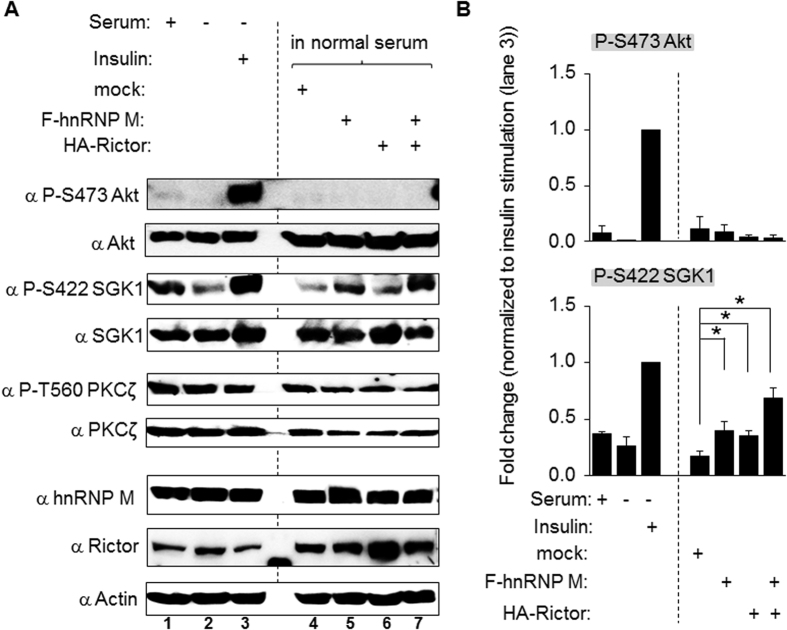
Overexpression of hnRNP M augments the phosphorylation of SGK1 S422. (**A**) C2C12 cells were transiently transfected with plasmids containing mock, FLAG tagged hnRNP M (F-hnRNP M), HA tagged Rictor (HA-Rictor), or both. Cell lysates were processed by a Western blot analysis in order to detect the corresponding proteins and the various related signaling molecules. The first three lanes (lane 1, 2, and 3) were cells treated with normal serum, cells subjected to serum withdrawal, or cells subjected to insulin stimulation after serum withdrawal and their signaling changes were used as positive controls for the antibodies. These blots are representative of one experiment that was repeated three times. (**B**) The level of protein phosphorylation of Akt S473 and SGK1 S422 on the blots was quantified using NIH Image J freeware. The y-axis is the percentage of the insulin stimulation result (panel A, lane 3), which was set to 1. Data are the mean ± SD (n = 3). *p < 0.05.

To verify that hnRNP M is involved in the SGK1 S422 phosphorylation, we also used a gain-of-function approach. C2C12 cells were transient transfected with FLAG tagged hnRNP M, HA tagged Rictor, or both plasmids together (Supplementary Fig. S2). As shown in the Fig. 4A, while the phosphorylation sites at Akt S473 and PKCζ T560 showed no changes, phosphorylation of SGK1 S422 was significantly increased when there was overexpression of FLAG-hnRNP M or overexpression of HA-Rictor. Furthermore, these increases were additive when both plasmids were co-transfected into the same cell. Within the same set of experiments, a variety of other conditions, such as the addition of serum, serum withdrawal, and insulin re-addition after serum withdrawal, were included to demonstrate the efficacy of antibodies (lane 1–3). A quantification of the results is presented in Fig. 4B. It is also worth noting that the basal level of Akt phosphorylation at S473 in C2C12 was not easily detectable and therefore we normalized all the band density against the insulin re-addition after serum withdrawal condition (lane 3). At the same time, unexpectedly, overexpression of Rictor was found to have no effect on the phosphorylation of Akt; this might have been due to the cell type used and/or the lack of large amounts of one or more component(s) needed to form significant amounts of the complex. The latter would result in no change in protein phosphorylation because there could be only a very limited increase in the amount of active complex in the cells.

### HnRNP M or Rictor knockdown decreases the efficiency of C2C12 differentiation

**Figure 5:**
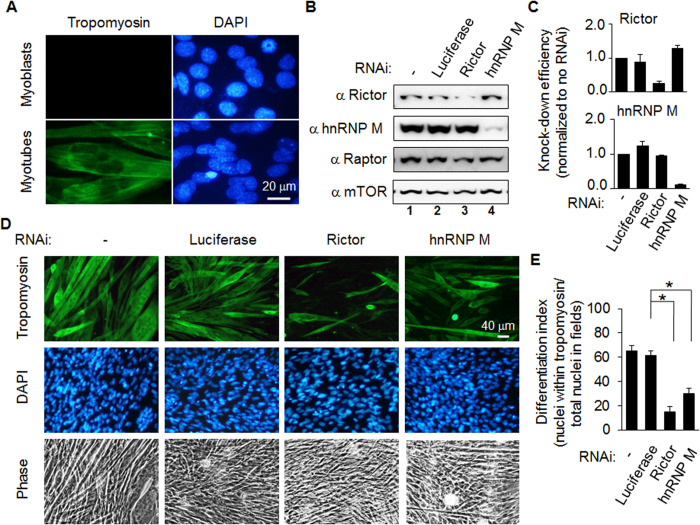
Differentiation of C2C12 myoblasts into myotubes is blocked by depletion of either Rictor or hnRNP M. (**A**) The level of myotubes differentiation can be determined using tropomyosin antibody as the primary antibody in an immunocytochemistry assay. In a parallel experiment, myoblasts were found to show no tropomyosin signal. The nuclei of the cells in the same field were stained with DAPI. (**B**) C2C12 myoblasts were infected with luciferase, Rictor, or hnRNP M RNAi viral particles, which was followed by puromycin selection. The cell lysates were processed and used Western blotting analysis to measure knock-down efficiency. (**C**) The abundance of Rictor and hnRNP M on the blots was quantified using NIH Image J freeware. The y-axis is the percentage of cells without any treatment, which was set to 1. Data are the mean±SD (n = 3). *p < 0.05. (**D**) C2C12 myoblasts were infected with luciferase, Rictor, or hnRNP M RNAi viral particles, which was followed by puromycin selection. The same number of cells was seeded onto each cover slip in order to initiate the differentiation process. Five days after differentiation had started, the cells were fixed and immunocytochemistry performed. (**E**) The differentiation index was calculated by counting the number of nuclei present in cells showing tropomyosin staining, which was then divided by total number of nuclei in the same field. The analysis was carried out using MetaMorph software. In total around 900 cells was quantified for each field. Data are the mean ± SD. *p < 0.05.

We further investigated the cellular functions associated with hnRNP M and the mTORC2 complex. Rictor has been implicated in the muscle differentiation^14^,^15^ and therefore we investigated the role of hnRNP M in the regulation of C2C12 differentiation. First we use tropomyosin, a bio-marker of mature muscle, to detect muscle differentiation (Fig. 5A). When the green images were merged with the blue DAPI stained images, we found that not all of the cells were stained with tropomyosin, which supports the idea that the antibody used is specific for tropomyosin. Figure 5B and C show the protein levels of the knockdown targets in the C2C12 myoblast cells and their quantification. It was found that Rictor and hnRNP M were dramatically decreased under the experimental conditions. Next, we examined the effect of depletion of Rictor on the level of C2C12 differentiation as compared to the luciferase RNAi group or cells without any treatment; depletion of Rictor decreased differentiation as expected. When C2C12 cells that had undergone hnRNP M knockdown were examined, it was found that the results were similar to those of the Rictor knockdown group (Fig. 5D). To quantify the level of the differentiation, the number of nuclei of cells with tropomyosin staining within each field was divided by total number of nuclei in the same field. Each field contained around 300 nuclei. The results showed that the level of differentiation of the parental C2C12 cells, the control RNAi cells, the Rictor knockdown cells, and the hnRNP M knockdown cells were 65% ± 5%, 62% ± 3%, 15% ± 4%, and 30% ± 5%, respectively (Fig. 5E). It should be noted that the initial seeding number of C2C12 myoblasts is one of the critical factors that affects the success of muscle differentiation. Therefore, we eliminated this variable by counting and seeding the same number of cells each time. This can be seen by comparing the density of DAPI staining across fields in Fig. 5D. In addition, we also noticed that cells undergoing differentiation became rhomboid in shape when there was Rictor or hnRNP M knockdown rather than forming multinucleate cells with a fiber shape, which was the situation with the controls. Taking the above results as a whole, the findings show that depletion of either Rictor or hnRNP M is able to inhibit the differentiation of C2C12 myoblasts.

### The reduction in muscle differentiation in Rictor knockdown cells is rescued by overexpression of hnRNP M

**Figure 6:**
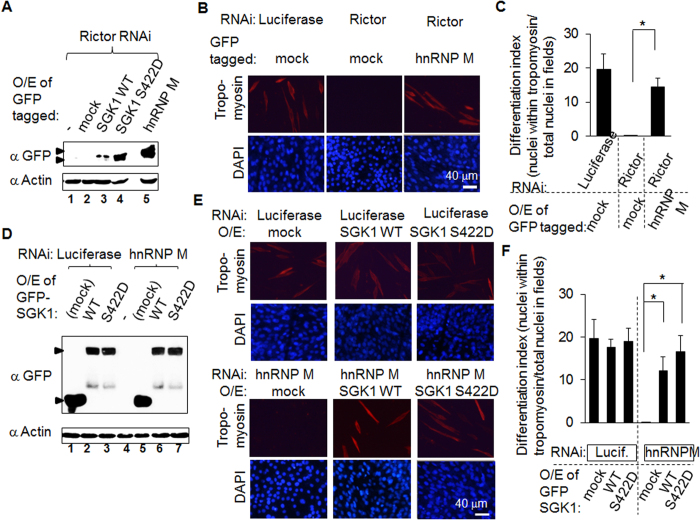
The decreases in differentiation of Rictor and hnRNP M knockdown C2C12 myoblasts are both rescued by overexpression of either hnRNP M or SGK S422D. (**A**) and (**D**) C2C12 myoblasts containing Rictor RNAi were infected with virus that contained the CMV promoter driving overexpression (O/E) of hnRNP M. After three days infection, cell lysate was processed by a Western blot analysis to detect GFP hnRNP M in (**A**) or GFP SGK1 in (**D**). (**B**) and (**E**) C2C12 myoblasts containing the indicated constructs began to differentiate into muscle. On day 5 after differentiation, a difference was observed between muscles containing hnRNP M and the control in (**B**) or between cells containing SGK1 WT/S422D and the control cells in (**E**). These samples were fixed and immunocytochemistry was performed using tropomyosin (red) as the primary antibodies and DyLight 549-conjugated anti-rabbit IgG as the secondary antibodies. The same field was also stained with DAPI (blue). (**C**) and (**F**) The differentiation index was calculated in the same way as described in Fig 5E. The images usually involved three different fields for each experiment, and each experiment was repeated three times. Each image typically contained more than 200 cells. Data are shown as the mean ± SD. *p < 0.05.

To verify the relationship between Rictor and hnRNP M, we utilize a rescue strategy. Viral particles containing a CMV promoter that constitutively drove GFP tagged hnRNP M overexpression was used to infect the Rictor knockdown C2C12 cells. The protein expression levels in these cells are presented in lane 5 of Fig. 6A. In the same experiment, viral particles containing a CMV promoter constitutively driving either GFP tagged SGK1 WT or S422D were also included (lane 3 and lane 4). When we investigates differentiation (Fig. 6B), it was found that two rounds viral infection led to significant decrease in the efficiency of differentiation (65% in Fig. 5E, which was decreased to 20% in Fig. 6C). If the control is taken as an example, the two-round of viral infection means that there was luciferase RNAi viral infection and puromycin selection during the first round, which was followed by viral infection containing the GFP tagged proteins during the second round. Under these conditions, C2C12 differentiation by the Rictor knockdown/GFP overexpression group is almost completely abolished. Furthermore, overexpression of hnRNP M in the Rictor knockdown cells results in the re-establishment of differentiation (Fig. 6B). The differentiation indices of the luciferase RNAi/GFP, Rictor RNAi/GFP and Rictor RNAi/GFP-hnRNP M cells were found to be 20% ± 4%, 0%, and 15% ± 2%, respectively (Fig. 6C).

### The decrease in muscle differentiation in the hnRNP M knockdown cells is rescued by overexpression of SGK1 S422D

To investigate whether differentiation is mediated via SGK1, we expressed the constitutive active form of SGK1, SGK1 S422D, in hnRNP M knockdown cells. The GFP blot shows the expression levels of GFP, GFP-SGK1 WT, and GFP-SGK1 S422D (Fig. 6D). As shown in the Fig. 6E, and as mentioned above, the use of two-round viral infection led to a significant decrease in differentiation efficiency with the differentiation of C2C12 cells in the hnRNP M knockdown/GFP overexpression group being almost completely abolished. As expected, overexpression of SGK1 S422D in the hnRNP M knockdown cells results in the re-establishment of differentiation. The differentiation indices were quantified for the luciferase RNAi/GFP, luciferase RNAi/GFP-SGK1 WT and luciferase RNAi/GFP-SGK1 S422D cells and were found to be 20% ± 4%, 18% ± 2%, and 19% ± 3%, respectively. Furthermore, the differentiation efficiency of the hnRNP M RNAi/GFP, hnRNP M RNAi/GFP-SGK1 WT and hnRNP M RNAi/GFP-SGK1 S422D cells were found to be 0%, 12% ± 3%, and 17% ± 4%, respectively (Fig. 6F).

### The decrease in C2C12 differentiation mediated by hnRNP M loss-of-function seems to be mediated via SGK1/FoxO axis signaling

**Figure 7:**
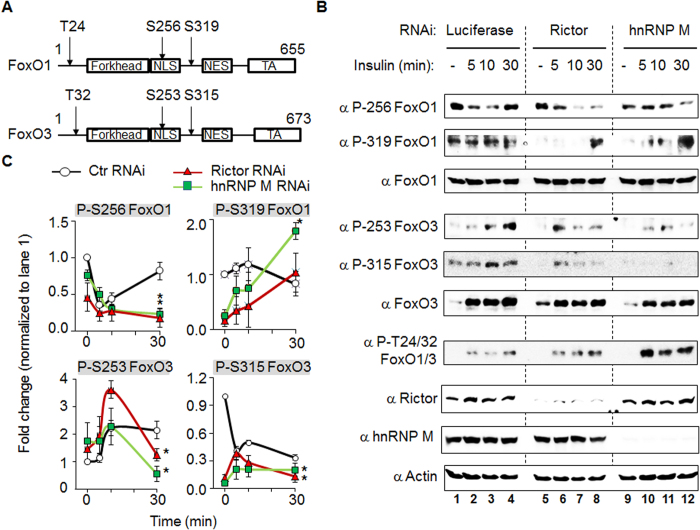
The phosphorylation of FoxO1 and FoxO3 is affected in insulin-treated C2C12 cells that contain either Rictor or hnRNP M RNAi. (**A**) A cartoon indicates the domain and the three key phosphorylation sites present on both FoxO1 and FoxO3. NLS, nuclear localization signal; NES, nuclear export signal; TA, transactivation domain. (**B**) C2C12 myoblasts infected with Rictor or hnRNP M RNAi were stably selected using puromycin. These cells were treated with 100 nM insulin at the indicated time. Lysates obtained from the insulin-treated cells were separated using SDS-PAGE, which was followed by Western blot analysis using the indicated antibodies. (**C**) Phosphorylation on these blots was quantified using NIH Image J freeware. In addition to the phosphorylation of FoxO3, the y-axis is the fold change in expression normalized against the insulin stimulation at 0 min (lane 1), which was set to 1. Since the levels of FoxO3 protein change over the time, the individual phosphorylation form has been normalized to corresponding total protein amount present in order to obtain a number at each time point. The number at each time point has then been further normalized against the one at time 0, which was set to 1. All blots are representative of one experiment that was repeated four times. Data are the mean±SD (n = 4). *p < 0.05. *vs*. luciferase RNAi at the same timing.

We further investigated in more detail the mechanism by which hnRNP M affects myotube differentiation. One molecule known to be involved with Akt and SGK1 in C2C12 differentiation is FoxO. FoxO is a common downstream protein of Akt and SGK1 and has previously be shown to be involved in the regulation of myoblast differentiation^16^. We examined the effect of hnRNP M on two FoxO proteins, namely FoxO1 and FoxO3 (Fig. 7A), because these two members of the FoxO family have been studied the most in muscle tissue^17^. As shown in Fig. 7B, phosphorylations of FoxO1 S256 and of FoxO3 S253/S315 were significantly decreased after insulin-treatment for 30 min in both the Rictor and hnRNP M RNAi cells. The quantified results are shown in Fig. 7C and these clearly demonstrate that all the sites, except FoxO1 S319, show the same change when Rictor or hnRNP M knock-down cells are used. These results suggest that Rictor/hnRNP M may mediate signaling via a number of downstream molecules, including SGK1 and the two FoxOs, and these signals then modify the biological functioning of the C2C12 cells.

## Discussion

In contrast to mTORC1, various aspects of mTORC2, namely its regulation, its downstream partners and its functions, are less well explored. In the present study we have carried out a set of strictly controlled immunoprecipitations involving Rictor wild-type and Rictor knockout MEF cells. This was done together with a proteomics analysis and we were able to identify hnRNP M as a novel binding partner of Rictor/mTORC2. HnRNP M is not only required for mTORC2 to phosphorylate SGK1 at S422, but also plays a crucial role in muscle differentiation. The mechanism of hnRNP M-mediated differentiation was found, plausibly, to be via the SGK1/FoxOs pathway.

Our findings indicate that hnRNP M binds to mTORC2, but not to mTORC1. HnRNP M is an hnRNP that is generally involved in RNA splicing^18^,^19^. While it is known to localize to the nucleus, it is also known to be a RNA-binding protein that undergoes nucleocytoplasmic shuttling during pre-mRNA splicing^20^. Thus, while it has been reported that mTORC1 is mainly localized within the cytoplasm, by way of contrast, mTORC2 has been found to be abundant in both the cytoplasmic and nuclear compartments^21^,^22^,^23^. This contrast might be the reason why we see only a fraction of the Rictor/mTOR in a cell associated with hnRNP M. Furthermore, if we explore the known functions of hnRNP M, these have been reported to be diverse. In addition to RNA splicing and localization to the nucleus, Jan *et al*. reported that hnRNP M is a target of poliovirus 3C proteinase and this activity then facilitates enterovirus infection^24^. Furthermore, utilizing pull-down and mass-spectrometry approaches, Luo *et al*. found that hnRNP M interacts with listeriolysin O and restricts the growth of *Listeria monocytogenes* in host cells^25^. HnRNP M has also been shown to be involved in cancer during invasion and metastasis, as well as being able to act as a biomarker for cancer^26^,^27^. Obviously, the functions of hnRNP M are likely to expand further in the future. Nevertheless, based on the protein’s ability to associate with RNA, it is important to know that hnRNP M binds to mTORC2 because this could have a multitude of implications when searching for RNA molecules that will bind to the hnRNP M/mTORC2 complex.

How can the hnRNP M/mTORC2 complex activate SGK1? We propose that the increased phosphorylation of SGK1 S422 that is brought about by overexpression of hnRNP M might be mediated via either an increased association with the substrate SGK1 or by an increased amount/activity of mTOR enzyme present in the cell. In our preliminary experiments we did not see any binding between SGK1 and hnRNP M at the basal level. However, it is plausible that more hnRNP M might bring more mTOR into the complex since hnRNP M associates with mTOR. Furthermore, ribosomes have been showed to play a direct role in activating mTORC2^28^. On the other hand, when associated with ribosomes, it has been shown that mTORC2 causes the phosphorylation of Akt at T450 and this stabilizes the Akt protein co-translationally^29^. Recently, Dai *et al*. have also reported that mTORC2 phosphorylates IGF-2 mRNA-binding protein 1 (IMP1) co-translationally too. Such an event eventually enhances the production of IGF2 protein, as well as promoting cell proliferation^30^. Therefore, whether the increased phosphorylation of SGK1 S422 that is brought about by overexpression of hnRNP M is mediated via the ribosome or via co-translation, needs to be investigated in the future.

Interestingly, while mTOR is ubiquitously expressed in a wide range of tissues, it has been found to have particularly high levels of expression in skeletal muscle and the kidney^31^. Hung *et al*. discovered recently that Rictor is dispensable during muscle regeneration *in vivo*; this was done by conditionally deleting Rictor in Myf5 precursors. By way of contrast, they found that Rictor is required for brown adipocyte differentiation *in vitro*^32^. Notwithstanding the above, several papers have argued the viewpoint experimentally that mTORC2 and/or mTORC1 play role(s) in the muscle differentiation^14^,^15^,^33^. Ge *et al*. found that, although mTOR knockdown severely impairs myogenic differentiation as expected, when there is knockdown of Raptor or Rheb there is enhancement of differentiation. Consistent with a negative role for these proteins in myogenesis, overexpression of Raptor or Rheb has been found to inhibit C2C12 differentiation^34^. Furthermore, downregulation of Rictor, but not downregulation of Raptor, has been shown to prevent terminal differentiation (fusion) of C2C12 myoblasts. These findings support the hypothesis that mTORC2 is critical to muscle differentiation. Specifically, in support of this, the expression of the phosphomimetic mutant Akt S473D in Rictor-deficient cells has been found to rescue myoblast fusion even in the presence of rapamycin^15^. In addition, it was found recently that hnRNP M is involved in promoter-dependent translation during myoblast differentiation^35^. Our findings underscore the versatility of mTOR signaling during biological regulation and we suggest that hnRNP M/Rictor are likely to have at least one or perhaps even more roles during skeletal myogenesis.

Based on the above, we also investigated the SGK1-FoxOs pathway as a target of mTORC2/hnRNP M during the regulation of muscle differentiation. It has been shown previously that FoxO1 and FoxO3 are involved in muscle differentiation regulation^17^. Furthermore, other reports have demonstrated that SGK1 is able to directly phosphorylate FoxO1 and FoxO3 at T24/S256/S319 and T32/S253/S315, respectively^36^. The FoxOs, which form the O subclass of the forkhead family, are transcription factors and are known to have a broad range of functions; they have been associated with the regulation of life span, the control of catabolic pathways, cell fate and myogenic differentiation^16^,^37^,^38^. Kamei *et al*. reported that overexpression of FoxO1 in skeletal muscle leads to reduced muscle mass, a down-regulation of type I fibers genes and an impairment of glycemic control^39^; however, the activation status of the transgenic FoxO gene was not determined in this content. Furthermore, it has been reported that a constitutively nuclear FoxO1, namely the de-phosphorylated form, inhibits differentiation of C2C12 cells from myoblasts to myotubes^16^. Thus it would seem that, when FoxO1 is phosphorylated, it becomes sequestered in the cytosol and unable to inhibit muscle differentiation. Furthermore, constitutively active FoxOs, when present in C2C12 myotubes, have been shown to enhance myostatin promoter activity^40^. To our knowledge, the present study is the first report indicating that hnRNP M is likely to be a mediator that is able to relay signaling from mTORC2 to SGK1 and possible the FoxOs and as a result control muscle differentiation.

In conclusion, we have identified a novel Rictor associated protein, hnRNP M, and found that hnRNP M is able to affect the phosphorylation of SGK1 S422, which is recognized as a direct phosphorylation site of mTORC2. However, hnRNP M has no effect on the phosphorylation of Akt S473, PKCα S657 or PKCζ T560. In addition, hnRNP M would seem to mediate the SGK1 signaling that regulates muscle differentiation. These findings emphasize the novel role that hnRNP M has in future research targeting the mTORC2 signaling pathway.

## Methods

### Reagents

The materials and reagents were purchased from the companies indicated in parentheses: Dulbecco’s modified Eagle’s medium (DMEM, Gibco-BRL-Life Technologies, NY, USA); fetal bovine serum (FBS, Thermo Scientific, Utah, USA); horse serum (Gibco-BRL-Life Technologies, NY, USA); protein G-agarose and protein A-agarose, antibodies against hnRNP M, p70 S6 kinase, SGK1 S422, PKCα S657, PKCζ and FoxO1 S319 (Santa Cruz Biotechnology, CA, USA); antibodies against mTOR, Rictor, Raptor, SGK1, PKCα, Akt1 S473, pan Akt1 and anti-GFP antibodies (Epitomics, CA, USA); antibodies against p70 S6 kinase T389, Akt T308, PKCζ T410 and FoxO1/FoxO3 T24/T32 (Cell Signaling Technology, MA, USA); antibodies against PKCζ T560, FoxO1, FoxO3, FoxO1 S256 and FoxO3 S253 (Abcam, Cambridge, UK); antibodies against FLAG and tropomyosin (Sigma, MO, USA); horseradish peroxidase anti-rabbit/anti-mouse secondary antibodies (Jackson, PA, USA); anti-rabbit/anti-mouse immunoglobulin G (IgG) antibodies conjugated to Alexa fluor 488 and anti-rabbit/anti-mouse immunoglobulin G (IgG) antibodies conjugated to DyLight 549 (Invitrogen, CA, USA); and Fluoromount G (Southern Biotech, AL, USA). The anti-FoxO3 S315 antibodies were a generous gift from Dr. Michael Greenberg (Harvard Medical School, Boston). All other chemicals were from Sigma (MO, USA) unless indicated otherwise.

### Plasmid construction

The wild-type or various mutants of *Rictor, hnRNP M, mSin1, mLST8, p53, SGK1, FoxO1* and *FoxO3* genes were subcloned into an appropriate mammalian expression vector to create proteins tagged with FLAG, HA, or GFP as required. Parts of hnRNP M, namely aa 1~195, aa 1–532, and aa 533~730, were subcloned into pCMV5-FLAG using the BamHI and NotI sites and then these constructs were used for the mapping experiment. Parts of Rictor, namely aa 1~860 and aa 1181–1708 were subcloned into pCMV5-HA using the BamHI and NotI sites, too. All constructions were confirmed by DNA sequencing.

### Cell culture, differentiation and transfection

HEK 293T cells and C2C12 myoblasts (Bioresource Collection and Research Centre, Taiwan) were cultivated in DMEM supplemented with 10% FBS and penicillin/streptomycin in 5% CO_2_ at 37 °C. For the muscle cell differentiation experiments, C2C12 myoblasts were seeded onto 6 cm plates until 95% confluency and then cultivated in DMEM containing 2% horse serum. We changed the medium every other day until C2C12 myoblasts had formed. When transfection was performed, HEK 293T cells were plated at a density of 3.5 × 10^6^ on 10 cm dishes and transfected 5 h later with a total of 8 μg of DNA and 30 μl of Lipofectamine per dish; the procedure followed the manufacturer’s instructions (Invitrogen, CA, USA). The DNA mixture was added drop by drop onto the cells in the 10 cm dish and the dish was then incubated for additional 36 to 48 h. Regarding transfection of C2C12 cells, which are from a so called “hard-to-transfect” cell line, we adopt the method described by Escobedo and Koh^41^. The transfection rate was still low at around ~35% as judged by GFP transfection.

### Cell lysates, immunoprecipitation, and Western blotting

Frozen cells was scraped from the dish into lysis buffer (20 mM Tris base, pH 7.9, 20 mM NaCl, 1 mM EDTA, 5 mM EGTA, 20 mM β-glycerophosphate, 1 mM dithiothreitol, 1 mM phenylmethylsulfonyl fluoride, 25 nM calyculin A, 0.25% (w/w) CHAPS, 1 tablet/50 ml of protease inhibitor (Roche Molecular Biochemicals)). Next, 2 μg of IgG, Rictor, Raptor or FLAG antibodies were mixed with protein G-agarose or protein A-agarose at 4 °C for 1 h and then washed three times in lysis buffer. Then the cell lysates were centrifuged at 13,500 rpm for 10 min and aliquots of the supernatants, containing equal amounts of protein as measured by Bradford assay (Bio-Rad), were added to 15 μl of settled FLAG-agarose beads and incubated at 4 °C for 2 h. The beads were washed three times with 1 ml of lysis buffer, twice with 1 ml of lysis buffer containing 0.5 M NaCl, and then once with 1 ml of lysis buffer. Finally the adsorbed proteins were released using SDS sample buffer and incubation at 95 °C for 10 min. The released proteins were separated by SDS-polyacrylamide gel electrophoresis, which was followed by transferring onto a polyvinylidene difluoride membrane. Each membrane was then probed with the antibodies as indicated. The blots were visualized using horseradish peroxide-conjugated secondary antibody followed by chemiluminescence using the manufacturer’s protocol (Millipore, MA, USA).

### Immunocytochemistry

C2C12 myoblasts were seeded onto glass slides. After reaching 90–95% confluence, the C2C12 cells were induced to differentiate. Once differentiated, the cells were fixed using 4% paraformaldehyde for 30 min and then permeabilized with 0.3% Triton X-100 in PBS for 10 min at room temperature. Next the samples were blocked with 3% bovine serum albumin (BSA) in PBS for 1 hour. The required primary antibodies, such as anti-tropomyosin, were then added for 2 h and this was followed by washing with PBS. Next the appropriate secondary antibody, such as Alexa 488-conjugated anti-mouse (Invitrogen) or DyLight 549-conjugated anti-mouse (Invitrogen), was incubated with the cells for 45 min and this was then followed by a PBS wash. Next the nuclei of the cells were stained with 4′, 6′-diamidino-2-phenyindole (DAPI) for an additional 15 min, which was followed by another PBS wash. Each coverslip was mounted on a glass slide using Fluoromount G (Southern Biotech, Birmingham, AL, USA). Images were usually captured across three different fields for each experiment. Each experiment was repeated two or three times. A Zeiss Axio Observer D1 microscope (Carl Zeiss, Jena, Germany) was used for the microscopy. The differentiation index was calculated by counting the number of nuclei present in cells showing tropomyosin staining in a given field divided by total number of nuclei in the same field; the analysis was carried out using either Metamorph software or Image-Pro Plus software. In total, six to eight images were captured. The figures were prepared using Adobe software.

### Construction of the plasmids overexpressing various proteins and RNAi viral particle preparation

We constructed the overexpression plasmids using methods that have been described previously^42^. Briefly, we replaced the U6 promoter of the pLKO.1 puromycin construct with the cytomegalovirus (CMV) promoter, which is a constitutive promoter. HnRNP M, SGK1 WT, and the constitutive active form of SGK1, namely SGK1 S422D, were separately engineered so as to be fused with the green fluorescent protein (GFP) encoded by the plasmid. By taking advantage of the pLKO.1 backbone, the infection rate, selection, and preparation of viruses would be the same as when one uses RNAi. The shRNAs targeting luciferase, Rictor and hnRNP M were purchased from the National RNAi Core Facility at the Academia Sinica. The functioning clones and the corresponding effective target sequences of Rictor and hnRNP M were verified and are listed as follows: luciferase sequence 5′-CCGGCGCTGAGTACTTCGAAATGTCCTCGAGGACATTTCGAAGTACTCAGCGTTTTT, mouse Rictor sequence 5′-CCGGGCCAGTAAGATGGGAATCATTCTCGAGAATGATTCCCATCTTACTGGCTTTTTG, mouse hnRNP M sequence 5′-CCGGGCTGAAGTTCTAAACAAGCATCTCGAGATGCTTGTTTAGAACTTCAGCTTTTTG. The RNAi plasmids were co-transfected with two other viral packaging plasmids, pCMV8.91 and pMD.G, into the HEK 293T cells. The virus-containing medium was collected after transfection for 40 h and concentrated by ultracentrifugation at 25,000 rpm for 2 h. The pellets were then resuspended and stored at −80 °C until use. We followed a protocol provided by the RNAi Core Facility of the Academia Sinica, Taipei, Taiwan to measure the RNAi titer (http://rnai.genmed.sinica.edu.tw/file/protocol/4_1_EstimationLentivirusTiterRIUV1.pdf).

### Statistical Analysis

Data are presented as the mean ± standard deviation (SD). The treatment effects were evaluated using a two-tailed Student’s t-test. A *p* value of less than 0.05 was considered to be statistically significant.

## Additional Information

**How to cite this article**: Chen, W.-Y. *et al*. Heterogeneous nuclear ribonucleoprotein M associates with mTORC2 and regulates muscle differentiation. *Sci. Rep.* **7**, 41159; doi: 10.1038/srep41159 (2017).

**Publisher's note:** Springer Nature remains neutral with regard to jurisdictional claims in published maps and institutional affiliations.

## Supplementary Material

Supplementary Figures
