# Supplementary material for: Heterogeneous nuclear ribonucleoprotein M associates with mTORC2 and regulates muscle differentiation
Source: Sci Rep. 2017 Jan 20;7:41159. doi: 10.1038/srep41159 (PMC5247691; doi:10.1038/srep41159)
Supplement: Supplementary Figures [file srep41159-s1.pdf]

Supplementary Data

Heterogeneous nuclear ribonucleoprotein M associates with mTORC2 and regulates muscle differentiation

Wei-Yen Chen, Chia-Lung Lin, Jen-Hua Chuang, Fu-Yu Chiu, Yun-Ya Sun, Mei-Chih Liang, and Yenshou Lin

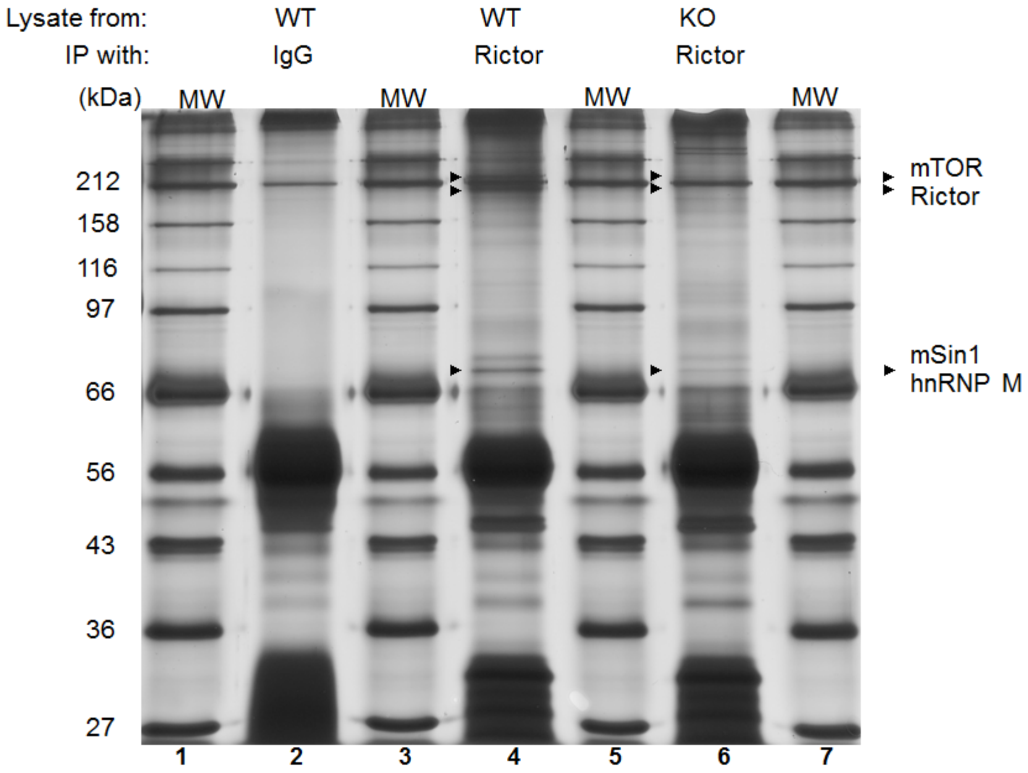

Supplementary Fig. S1. The candidates of the mTOR complex 2 present in the immunoprecipitation (IP) complex using Rictor wild-type (WT) vs. Rictor knockout (KO) mouse embryonic fibroblasts (MEFs). Due to the low survival rate of the Rictor KO MEFs, equal amounts of protein (1 mg) from the cell lysates of Rictor WT vs. KO MEFs were subjected to IP using 2 μg of Rictor antibody for each sample. The same amount of rabbit normal IgG with the same format was used as the control and loaded onto lane 2. The complex was then separated by 8% SDS-PAGE and the gel stained with Coomassie blue followed by Silver staining. The region around Rictor (180 kDa and 250 kDa, as indicated as arrows), which was compressed due to the percentage of the SDS-PAGE used, was verified to contain Rictor and mTOR by mass spectrometry analysis. The band indicated by arrows around 70 kDa was found to contain five proteins and these included mSin1 and hnRNP M.

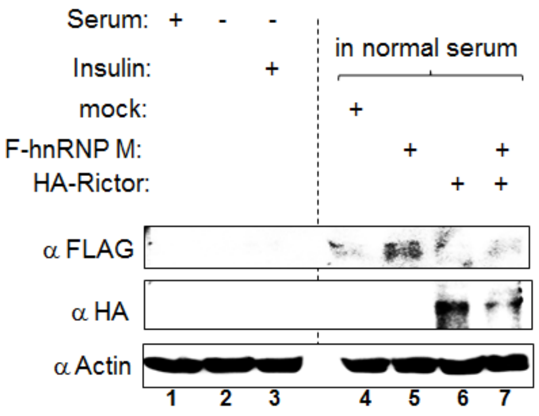

Supplementary Fig. S2.The expression of FLAG tagged hnRNP M, HA tagged Rictor, or both plasmids together in C2C12 cells. The C2C12 cells are a so called “hard-to-transfect” cell line. Even after we adopted the method described by Escobedo and Koh (Escobedo and Koh (2003) BioTechniques 35:936-940), the transfection rate was still quite low at ~35%, as judged by GFP transfection. In this experiment, we used a total 8 μg DNA for each condition with the cells grown in 10 cm cell culture plates, that is lane 5 with 8 μg DNA of FLAG-hnRNP M, lane 6 with 8 μg DNA of HA-Rictor, and lane 7 with 4 μg DNA FLAG-hnRNP M plus 4 μg HA-Rictor. Furthermore, we carried out separate blots using FLAG and HA antibodies to examine the expression of recombinant hnRNP M and recombinant Rictor in these C2C12 cells. We also use hnRNP M and Rictor antibodies to blot the same membrane in order to determine the difference between the endogenous and exogenous proteins and this is present in the Fig. 4A.
